# Automated Extraction of Key Entities from Non-English Mammography Reports Using Named Entity Recognition with Prompt Engineering

**DOI:** 10.3390/bioengineering12020168

**Published:** 2025-02-10

**Authors:** Zafer Akcali, Hazal Selvi Cubuk, Arzu Oguz, Murat Kocak, Aydan Farzaliyeva, Fatih Guven, Mehmet Nezir Ramazanoglu, Efe Hasdemir, Ozden Altundag, Ahmet Muhtesem Agildere

**Affiliations:** 1 Department of Medical Informatics, Faculty of Medicine, Baskent University, Ankara 06790, Türkiye; zaferakcali@yahoo.com; 2 Division of Medical Oncology, Department of Internal Medicine, Faculty of Medicine, Baskent University, Ankara 06790, Türkiye; 3 Department of Radiology, Faculty of Medicine, Baskent University, Ankara 06790, Türkiye

**Keywords:** mammography, prompt engineering, machine learning, named entity recognition (NER), natural language processing (NLP), radiology reports, clinicomics

## Abstract

Objective: Named entity recognition (NER) offers a powerful method for automatically extracting key clinical information from text, but current models often lack sufficient support for non-English languages. Materials and Methods: This study investigated a prompt-based NER approach using Google’s Gemini 1.5 Pro, a large language model (LLM) with a 1.5-million-token context window. We focused on extracting important clinical entities from Turkish mammography reports, a language with limited available natural language processing (NLP) tools. Our method employed many-shot learning, incorporating 165 examples within a 26,000-token prompt derived from 75 initial reports. We tested the model on a separate set of 85 unannotated reports, concentrating on five key entities: anatomy (ANAT), impression (IMP), observation presence (OBS-P), absence (OBS-A), and uncertainty (OBS-U). Results: Our approach achieved high accuracy, with a macro-averaged F1 score of 0.99 for relaxed match and 0.84 for exact match. In relaxed matching, the model achieved F1 scores of 0.99 for ANAT, 0.99 for IMP, 1.00 for OBS-P, 1.00 for OBS-A, and 0.99 for OBS-U. For exact match, the F1 scores were 0.88 for ANAT, 0.79 for IMP, 0.78 for OBS-P, 0.94 for OBS-A, and 0.82 for OBS-U. Discussion: These results indicate that a many-shot prompt engineering approach with large language models provides an effective way to automate clinical information extraction for languages where NLP resources are less developed, and as reported in the literature, generally outperforms zero-shot, five-shot, and other few-shot methods. Conclusion: This approach has the potential to significantly improve clinical workflows and research efforts in multilingual healthcare environments.

## 1. Introduction

Healthcare record-keeping has undergone a dramatic shift from paper-based systems to electronic medical records (EMRs), improving data management. However, the unstructured nature of much of the data within EMRs presents ongoing challenges for extracting clinically relevant information. While “big data” management was an initial focus, the field is increasingly turning to artificial intelligence (AI), particularly NLP, to glean meaningful insights from these data.

Named entity recognition (NER) in extracting key information from clinical texts, supporting tasks such as automated diagnosis coding, clinical decision support, and research. However, several challenges hinder the development and deployment of robust clinical NER systems. These challenges include the ambiguity and lack of standardized terminology in medical writing, with physicians often using abbreviations, acronyms, and varying styles depending on their training [1]. Furthermore, the contextual nature of clinical language, where the meaning of terms can change depending on the surrounding text, especially in negative findings, poses significant difficulty for accurate NER [1]. Developing clinical NER systems for languages other than English presents further linguistic and technical complexities [2,3].

Despite the advancements in NLP and the availability of large language models, automated information extraction from clinical texts, particularly for languages other than English and for specialized domains like mammography, remains a significant challenge. Existing clinical NER systems often rely on limited-context models, require extensive language-specific annotated datasets, or are not readily adaptable to the nuances of different medical report types. This lack of robust and adaptable solutions hinders the efficient utilization of valuable information locked within unstructured clinical text, particularly in multilingual healthcare settings.

Open-source NLP libraries like spaCy accessd on (https://spacy.io accessed on 23 December 2024) provide pre-trained models for several languages, including some designed for medical NER [3]. While these models offer a solid foundation, their performance can be enhanced by integrating transformer-based architectures. The Bidirectional Encoder Representations from Transformers (BERT) model, for instance, leverages attention mechanisms to effectively capture relationships within text, leading to improvements in NER accuracy [2]. Generative pre-trained transformer (GPT) models, another variant of the transformer architecture, are particularly well-suited for text generation tasks. A simple example demonstrating NER using spaCy with the GerNERMed model (for German) is shown below:
import spacy nlp = spacy.load(“de_GERNERMED”) doc = nlp(“Dem Patienten wurde die Einnahme von Paracetamol (500 mg, zwei Tabletten täglich, 8 Wochen lang) zur Behandlung empfohlen”.) # Show entities print(doc.ents)

LLMs, built upon the GPT architecture, such as OpenAI’s GPT-4, Google’s Gemini Pro, Anthropic’s Claude, Meta’s LLAMA, and Mistral’s Mixtral, have greatly expanded NLP’s capabilities. These models can handle diverse tasks, including NER, summarization, translation, and classification, without extensive task-specific training on GPUs. A key advantage of LLMs is their capacity for few-shot or even zero-shot learning, adapting to new tasks with minimal or no labeled examples. This distinguishes them from traditional NLP models. However, LLMs have limitations: they can be slower than specialized frameworks like spaCy and incur costs when accessed via cloud-based APIs [4]. Despite these limitations, LLMs generally outperform traditional spaCy models (without pre-trained transformers) on NER tasks [5].

Biomedical LLMs like BioBERT, fine-tuned on medical literature, have further advanced clinical NER [6]. This fine-tuning process enhances the models’ understanding of medical terminology and context. However, creating these specialized models requires substantial language-specific annotated datasets, which are often unavailable [7]. For example, a French equivalent of BioBERT would need a large, annotated French biomedical corpus. In contrast, GPT models require less language-specific data for adaptation, offering a more scalable approach to multilingual clinical NER. Retrieval-augmented generation (RAG) can efficiently incorporate external knowledge into LLMs, especially those with limited context windows. However, recent, large-context LLMs like Gemini and GPT-4 can directly process lengthy texts (e.g., Gemini 1.5 Pro handles up to 1.5 million tokens). Although long-context LLMs excel at handling extended text, RAG remains more cost-effective [8].

LLMs like GPT-4 and Gemini Pro can be prompted for NER tasks using varying numbers of examples, influencing the learning approach. Zero-shot learning provides no examples, relying solely on the model’s pre-training and the prompt itself. One-shot learning offers a single example, as illustrated below:
Extract the named entities from the following sentences in the medical domain: Example: Sentence: “The trachea and main bronchi have been evaluated as having normal configuration.” Entities: [trachea, main bronchi, normal configuration] Now, extract the named entities from this sentence: “The liver shows no signs of cirrhosis or hepatomegaly.” Output: [liver, cirrhosis, hepatomegaly]

Few-shot learning incorporates a limited number of examples (e.g., five), further clarifying the task. Many-shot learning provides a larger set of examples to enhance performance, though input length constraints can limit the number used.

This study addresses these challenges by introducing a novel prompt engineering approach for extracting key entities from mammography reports, specifically focusing on the Turkish language. Our contributions are as follows:

1- Development of a highly specialized, many-shot prompt: We introduce a systematically developed prompt (1195 lines, 165 examples (shots), 26,000 tokens) specifically designed for the nuances of mammography reports. This prompt incorporates comprehensive annotation guidelines, numerous examples covering a wide range of clinical scenarios, and specific instructions to handle the challenges unique to this domain, such as distinguishing between presence, absence, and uncertainty of findings. The prompt’s size and complexity are essential for capturing the intricate language, subtle variations, and domain-specific terminology found in these reports.

2- Adaptability to non-English languages: Unlike most existing clinical NER work that focuses on English, our prompt is designed for adaptability to other languages. We demonstrate its effectiveness with Turkish mammography reports, highlighting its potential for broader application in multilingual clinical information extraction.

3- Leveraging a large context window LLM with a generous free tier: We utilize Gemini 1.5 Pro, chosen for its combination of a large context window (crucial for handling long, complex reports), a generous free tier (facilitating accessibility and experimentation), and strong performance in preliminary evaluations. This allows researchers to explore and adapt our prompt without incurring immediate costs.

4- Demonstrated high accuracy in mammography report annotation: We demonstrate the effectiveness of our approach in accurately annotating Turkish mammography reports, achieving high F1 scores for key entities (ANAT, IMP, OBS-P, OBS-A, OBS-U).

By addressing the limitations of existing approaches, particularly for non-English languages and specialized medical domains, this study provides a significant advancement in clinical information extraction.

The F1 score, a crucial metric in NLP, assesses the performance of tasks by considering both precision and recall. More simply, the F1 score measures a model’s accuracy by balancing precision (the proportion of correctly identified entities among all predicted entities) and recall (the proportion of correctly identified entities among all actual entities). It ranges from 0 to 1, with 1 indicating perfect performance. Calculated as the harmonic mean of precision and recall (F1 = 2 * (Precision * Recall)/(Precision + Recall)) [1], the F1 score ranges from 0 to 1, where 1 represents perfect performance. Exact match F1 scores require precise agreement on entity type, boundaries, and span, while relaxed match F1 scores prioritize correct entity type and overlapping boundaries.

The remainder of the paper details the related works, methodology, results, and a discussion of the implications of this work.

## 2. Related Works

Prior research has explored various prompting techniques for clinical NER, including zero-shot, few-shot [9,10,11], and instruction fine-tuning [11]. However, our approach uniquely leverages many-shot prompting with a large context window, a capability made recently accessible through models like Gemini 1.5 Pro. This distinction is crucial and represents a significant advancement in the field.

While studies such as the one on Chinese Medical NER [9] explore zero-shot and few-shot prompting in a practical application, they do not address the many-shot regime that our work investigates. Similarly, a review by Cheng [10] on LLMs in pathology, while discussing zero-shot and few-shot prompting, does not explore the potential of providing a large number of examples within an extended context window, focusing instead on summarizing the existing applications of LLMs in the field. Furthermore, older models, such as the one used in the study named “Few-Shot Learning for Clinical Natural Language Processing Using Siamese Neural Networks: Algorithm Development and Validation Study” [11], which investigated a limited number of shots (up to 16) using the GPT-2 model, do not explore the many-shot approach.

Our literature search on PubMed, using the query “many shot prompt” further confirms the limited research specifically focusing on many-shot prompting in the clinical domain, especially for specialized tasks like mammography report annotation. The ability to provide a large number of examples (165 in our case) within a single prompt, facilitated by Gemini 1.5 Pro’s current 1.0, 1.5, or recently released 2 million token context window, is a recent advancement. This allows the model to learn intricate patterns and nuances specific to the task and domain, leading to improved accuracy, as demonstrated in our results.

A recent study investigated prompt engineering with few-shot learning, comparing the performance of GPT-3.5 and GPT-4 against the BioClinicalBERT model [12]. Using zero-, one-, and five-shot prompts on the MTSamples dataset, GPT-4 achieved exact match and relaxed match F1 scores of 0.593 and 0.861, respectively, with five-shot prompting. BioClinicalBERT [13], however, achieved higher F1 scores of 0.785 (exact) and 0.901 (relaxed) in the same study. These findings highlight the potential of few-shot learning while also indicating the need for further refinement to reach the performance levels of specialized, pre-trained models. The prompting techniques described in Table 3 of that study [14], particularly when scaled to include a larger number of examples, offer a promising approach. This approach involves using HTML tags to mark entities (e.g., <span class = “OBS-P”>nodular opacities</span>, <span class = “ANAT”>right breast</span>), constructing prompts with sections for task description, entity markup guidance, definitions, and annotation guidelines. Example input–output pairs (up to five) are included at the end of the prompt, with a maximum template length of 5419 characters or 1155 tokens [12].

LLMs are typically accessed through cloud-based interfaces. One common method is via a web-based user interface, such as those provided by OpenAI’s ChatGPT (https://chatgpt.com, accessed on 23 December 2024) and Google’s AI Studio (https://aistudio.google.com, accessed on 23 December 2024), where users submit prompts and receive model-generated text. Alternatively, developers offer APIs that enable direct integration of LLM functionality into applications, automating prompt submission and response retrieval. This API-driven approach enhances scalability and efficiency, particularly for high-volume processing [12].

LLMs process text by dividing it into fundamental units known as tokens, representing words, sub-word components, or punctuation. These tokens are then converted into numerical representations, called token embeddings, which capture semantic meaning and contextual relationships. The RadGraph system, for example, uses a specific entity framework encompassing four main categories: Anatomical Structures (e.g., “axilla”) and Observations, which are further classified as Present, Uncertain, or Absent. Observations can include visual characteristics, physiological processes, or disease classifications (e.g., “calcifications”) [15].

Effective training of NER models for medical texts relies on substantial, accurately labeled datasets. Tools like the open-source Doccano platform [16,17] assist in this annotation process. However, achieving high accuracy often requires manual annotation of numerous reports by medical specialists, including radiologists and clinicians.

## 3. Methods

This study evaluated the effectiveness of NER using prompt-based learning with Google’s Gemini 1.5 Pro, a LLM with a 1.5-million-token context window. Our objective was to automatically extract key clinical entities from anonymized Turkish mammography reports. We adopted a many-shot learning approach, providing the model with 165 examples (shots) along with a comprehensive prompt. The total length of the prompt, including the examples, was 26,000 tokens, maximizing the usable context window for both Gemini 1.5 Pro and ChatGPT-4 Turbo. Initial testing revealed OpenAI’s ChatGPT-4 Turbo (free web interface version) to be unsuitable due to performance limitations and its restricted context window (32,768 tokens).

Our primary objective was to develop an effective prompt engineering strategy for accurate annotation of mammography reports. While various LLMs could potentially be used with this approach, initial qualitative assessments using the free-tier ChatGPT interface (context window limited to 32,768 tokens at the time of testing) revealed a higher rate of apparent mis-annotations compared to Gemini 1.5 Pro, even though the prompt fit within the available context window (26,000 tokens). This observation, combined with the need for a larger context window for accurate experimentation and broader accessibility provided by Gemini’s free tier, led us to select Gemini 1.5 Pro as the LLM for developing and evaluating our prompt engineering approach. The focus of this study is on the prompt design itself, and a comprehensive comparison across different LLMs is beyond the scope of this work. However, we acknowledge that exploring the prompt’s performance with other LLMs, such as Mistral Large, LLaMA 3, and Claude 3/3.5, represents a promising avenue for future research.

**Figure 1:**
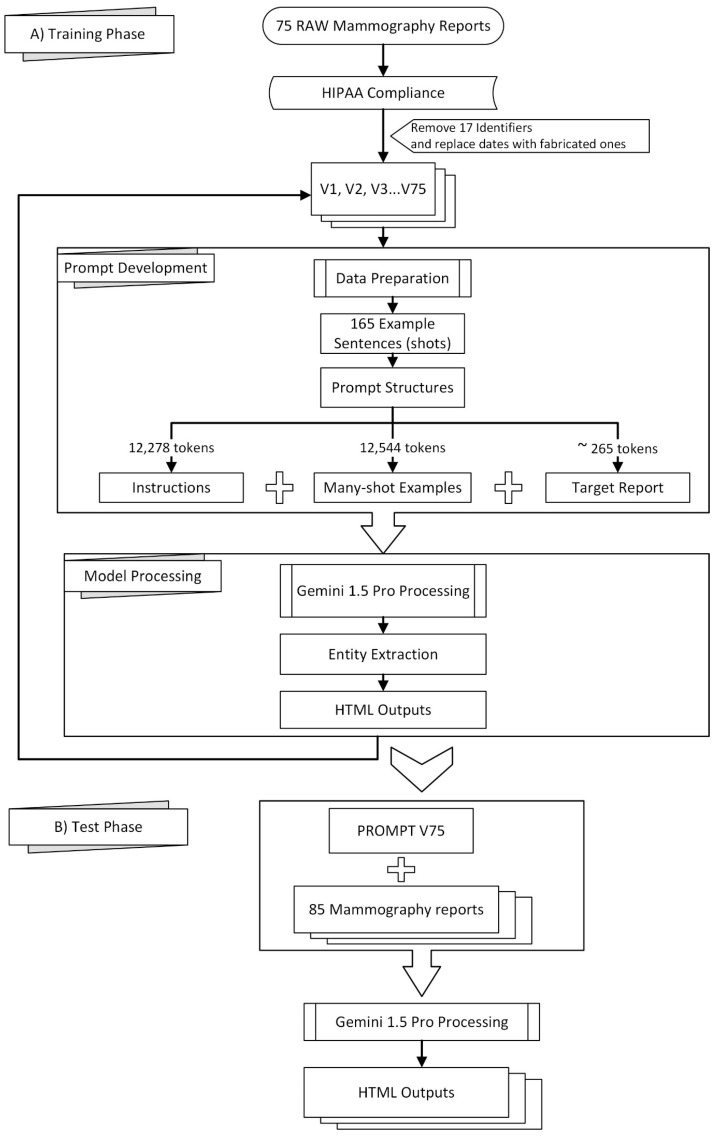
Framework of the methodology.

Figure 1 provides a visual overview of the methodology employed in this study, which can be broadly divided into a training phase (A) and a testing phase (B).

To aid in NER data processing, we developed the NER-tools GitHub repository [18], which contains a collection of Python scripts designed for various tasks. These scripts include the following:

- *jsonl2html.py* This script is used to convert JSONL files generated by Doccano into HTML files for visual inspection.
- *jsonl2spacy.py* This tool transforms Doccano’s JSONL files into the JSON format compatible with spaCy.
- *spacy2docbin.py* It converts files from spaCy’s JSON format into the binary DocBin format used for training.
- *docbin2jsonl.py* This script reverses the process, converting a spaCy DocBin file back into Doccano’s JSONL format
- *combine-htmls.py* Used for consolidating multiple HTML files into a single file.
- *html2jsonl.py* This script takes an HTML file and converts it back into Doccano’s JSONL format.
- *bio_converter.py* It processes the entities_f1.csv file, splitting it into y_pred.bio and y_true.bio for evaluation.
- *seqevalF1.py* Leverages the seqeval library to calculate performance metrics from y_pred.bio and y_true.bio files, as presented in Table 1.
- *relaxedF1.py* This script computes the relaxed F1 scores and generates the confusion matrix from the entities_f1.csv file (see Table 1 and Table 2).
- *patch-to-scorer.py*, A modified version of the get_ner_prf() function within spaCy’s scorer.py (v3.7.5), allowing for a more comprehensive set of metrics to be outputted. This modified function is designed to be placed in the C:\Python\Python311\Lib\site-packages\spacy directory.

To convert multiple html files to spacy docbin format, one must run scripts sequentially:

*combine-htmls.py* -> *html2jsonl.py* -> *jsonl2spacyjson.py* -> *spacy2docbin.py*

The sensitivity of spacy2docbin.py to punctuation marks adjacent to entities necessitates detaching these marks during data preprocessing. For example, “atelectasis -infarction” needs to be separated into “atelectasis “, “-”, and “infarction”. This approach is crucial only if you need to calculate exact-match F1 scores, as spaCy’s tokenization heavily relies on these precise distinctions for accurate evaluation.

Radiology reports from our university hospital are obtained in text format and manually edited to comply with HIPAA regulations, ensuring patient privacy Table 1 of [19]. Seventeen identifiers are removed from each report before being sent to the remotely hosted Gemini 1.5. As the eighteenth identifier, dates are either removed entirely or replaced with fabricated ones.

Preprocessing of the mammography reports included the removal of 17 identifiers to comply with HIPAA regulations, following the guidelines outlined in Table 1 of [19], which were also either removed or replaced as the 18th identifier. We received complimentary private preview access to Gemini 1.5 Pro in February 2024, allowing prompt development and testing throughout March and April 2024. Using Google AI Studio’s web interface, we curated 165 example sentences from 75 mammography reports, guided by clinicians and radiologists specializing in mammography who identified significant sentences for effective model training. The resulting prompt was evaluated on a separate set of 85 reports using the v1beta version of Google’s JavaScript/Node.js Generative AI API, as the “v1beta” parameter required for accessing the Gemini 1.5 Pro preview was not available through the Python API at the time [20].

The prompt structure consisted of three parts: instructions (12,278 tokens), many-shot examples (12,544 tokens encompassing 165 examples), and the target report (approximately 265 tokens). See the Supplementary Materials for excerpts of these instructions. A transitional phrase, “Here is the mammography report to be tagged:”, preceded each target report. To ensure consistent output in Google AI Studio, control phrases were added to enforce code snippet formatting.

To ensure consistent output formatting when utilizing the Google AI Studio interface, two additional control phrases are included at the end of the report:
### Do not forget to output in a code snippet window. ### Why didn’t you output in a code snippet window?

These control phrases ensure to generate the model’s response within a designated code snippet format, enhancing output consistency and readability.

**Figure 2:**
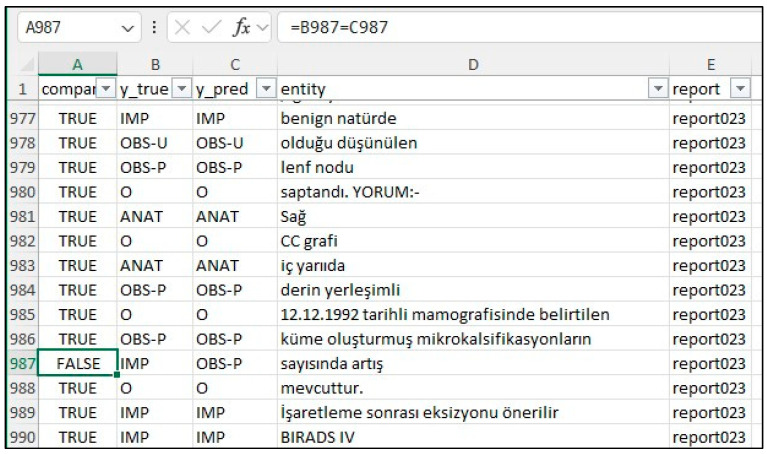
Example of manual annotation correction in Microsoft Excel for calculating relaxed F1 scores (shows the y_true column being edited.).

Our focus was on five key entities: anatomy (ANAT), impression (IMP), observation presence (OBS-P), absence (OBS-A), and uncertainty (OBS-U). Unannotated tokens (O) were also included in the evaluation. To calculate relaxed match F1 scores, we extracted entities from the Gemini 1.5 Pro HTML outputs using extract.py and merge.py [20], creating a merged CSV file. This file was then converted to an Excel file (entities_f1.xlsx) and reviewed by clinicians and radiologists specializing in mammography who corrected any annotation errors (Figure 2). If the model’s prediction was incorrect, the y_true column was edited, and the corresponding “Compare” column was marked as FALSE. The entities_f1.csv file was then used for subsequent analysis.

Creating prompts to prevent the model from annotating common non-entity phrases (e.g., “tarihli sağ dijital mamografi görüntüleri üzerinden yapılan karşılaştırmada” and “söz konusu olup”) proved challenging. Despite including duplicate examples with slight variations to reinforce this instruction, occasional mis-annotations required post-processing using rule-based corrections implemented in the following JavaScript code snippet:
const mistakes = [ ‘<span class = “ANAT”>Sol memenin</span> MLO ve CC mammogramları elde olunmuştur’, ‘Sol memenin MLO ve CC mammogramları elde olunmuştur’, ‘<span class = “ANAT”>Sağ memenin</span> MLO ve CC mammogramları elde olunmuştur’, ‘Sağ memenin MLO ve CC mammogramları elde olunmuştur’, ‘<span class = “ANAT”>SAĞ</span> MAMMOGRAFİ RAPORUNUN DA OKUNMASI ÖNERİLİR’, ‘SAĞ MAMMOGRAFİ RAPORUNUN DA OKUNMASI ÖNERİLİR’, ‘<span class = “ANAT”>SOL</span> MAMMOGRAFİ RAPORUNUN DA OKUNMASI ÖNERİLİR’, ‘SOL MAMMOGRAFİ RAPORUNUN DA OKUNMASI ÖNERİLİR’,  ];

Python code reads the entities_f1.csv file and prints relaxed F1 scores, and it is described below.
import pandas as pd from sklearn.metrics import classification_report  Read the CSV file df = pd.read_csv(“entities_f1.csv”, sep = “;”) # Extract the y_predict and y_true columns as arrays y_pred = df[“y_pred”].values y_true = df[“y_true”].values # Print the arrays (optional) print(“y_pred:”, y_pred) print(“y_true:”, y_true) print(classification_report(y_true, y_pred))

Python scripts read the entities_f1.csv file, output metrics, and confusion matrix using sklearn.metrics’s classification_report library (Table 1). The bio_converter.py Python script reads the entities_f1.csv file and converts it to y_pred.bio and y_true.bio files. The seqevalF1.py script reads y_pred.bio and y_true.bio files and outputs calculated metrics by using the seqeval library [21] (Table 1).

To calculate exact F1 scores, we trained a spaCy model using a pipeline optimized for accuracy. First, annotated HTML files were converted into the spaCy Docbin format, producing separate training (train.spacy) and testing (test.spacy) datasets (https://github.com/zakcali/GPT-MMG, accessed on 23 December 2024). The model was trained using the “dbmdz/bert-base-turkish-cased” transformer, selected within a configuration file specifying Turkish language and GPU hardware acceleration. An NVIDIA RTX 3090 GPU was utilized for accelerated training (https://spacy.io/usage/training#config, accessed on 23 December 2024).

The following command trains a spaCy model using 85 Gemini-annotated reports (test.spacy) as the training data and 75 human-annotated reports (train.spacy) as the development data. Note that the --paths.train and --paths.dev arguments are intentionally swapped to reflect this non-standard usage of the datasets. The command to train the model is as follows:
*python3 -m spacy train config.cfg --gpu-id 1 --output ./outputt --paths.train ./data/test.spacy --paths.dev ./data/train.spacy*

Because spaCy’s default evaluation does not provide metrics for unannotated entities or a confusion matrix, we patched the scorer.py file of spaCy V3.7.5 to collect tp, fp, fn, accuracy, and support values (Table 3). Model evaluation was performed using the spacy benchmark accuracy command.

The following command evaluates the model trained with Gemini-annotated reports (test.spacy) against human-annotated reports (train.spacy):
*python3 -m spacy benchmark accuracy .\output\model-best\ .\data\train.spacy --output metrics.json --gpu-id 0*

## 4. Results

**Figure 3:**
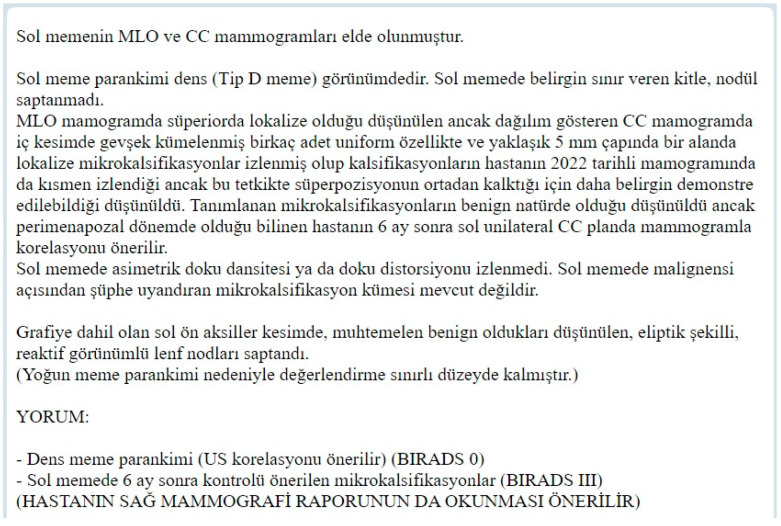
Example of a raw (unannotated) Turkish mammography report used as input for the LLM.

**Figure 4:**
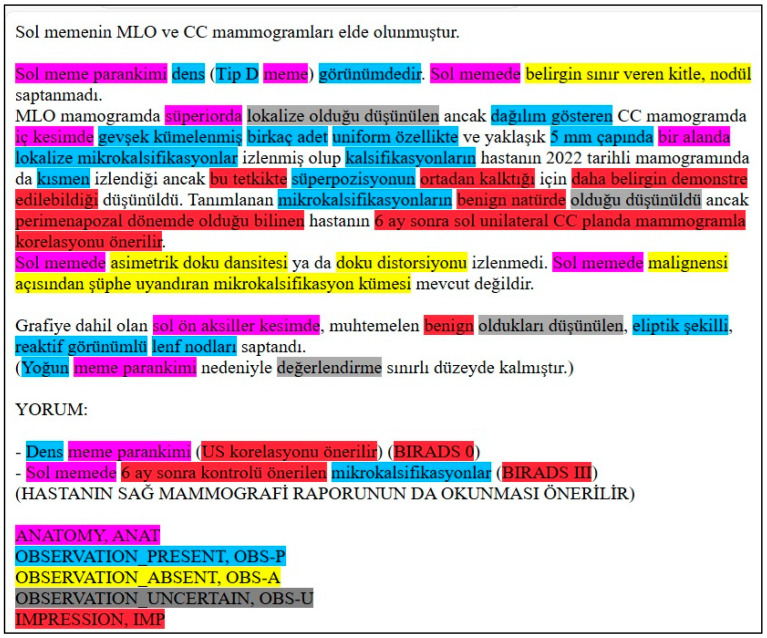
Annotated Turkish mammography report output from the LLM, displayed in HTML format.

**Figure 5:**
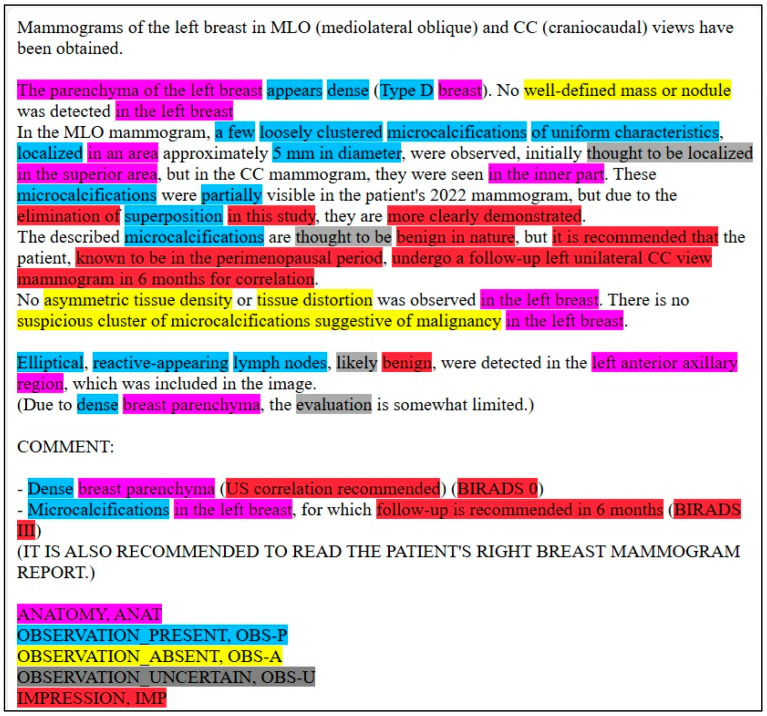
English translation of the annotated Turkish mammography report provided for reader convenience.

Figure 3 displays an example of the model’s unannotated raw input. Figure 4. displays an example of the model’s annotated output, and Figure 5 provides the corresponding English translation. The LLM performs analysis directly on the Turkish text. This translation is not used in the analysis process.

Evaluation of the test set using relaxed matching demonstrated excellent performance. The model achieved an overall accuracy of 0.99 and a macro-averaged F1 score of 0.99. Perfect F1 scores of 1.00 were obtained for both observation presence (OBS-P) and observation absence (OBS-A) entities, indicating high precision and recall in identifying these categories (see Table 1, Table 2 and Table 3). The model also performed well in recognizing anatomical entities (ANAT), impression (IMP), and uncertainty observations (OBS-U), with F1 scores of 0.99, 0.99, and 0.99, respectively.

Using exact matching, the overall accuracy was 0.71, and the macro-averaged F1 score was 0.84. The individual F1 scores for exact match were as follows: ANAT (0.88), IMP (0.79), OBS-P (0.78), OBS-A (0.94), and OBS-U (0.82).

**Figure 6:**
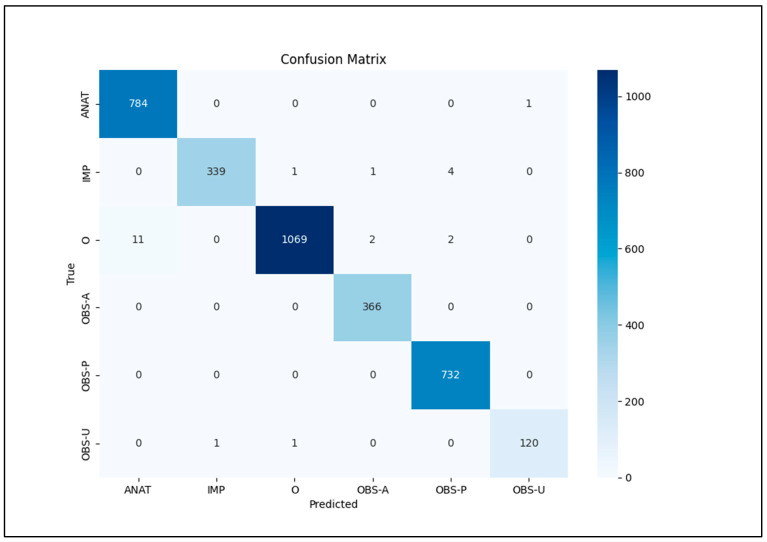
Relaxed match recognition confusion matrix.

The confusion matrix for relaxed match recognition is shown in Table 2 and Figure 6.

## 5. Discussion

The healthcare field faces challenges managing unstructured text data within relational SQL databases. While common in healthcare, SQL databases struggle with unstructured data, semantic interoperability, scalability, security, and integration with modern technologies like AI. This limits access to valuable information for patient care and research. Converting anonymized, SQL-stored medical data (compliant with GDPR/HIPAA) into formats like JSON/XML can facilitate AI processing. AI models trained for NER and classification could be instrumental in this data transformation. While current medical LLMs primarily learn from textbooks and published research, increasing efforts focus on incorporating de-identified patient data for improved real-world performance.

The 2017 paper “Attention is All You Need” [22] introduced the Transformer architecture, revolutionizing NLP. Pre-transformer methods relied on libraries like NLTK, a precursor to spaCy. Transformers enabled powerful models like BERT (Bidirectional Encoder Representations from Transformers) and GPT (Generative Pre-trained Transformer). While both leverage the Transformer architecture, they differ in function: BERT excels at understanding context for tasks like text classification, while GPT focuses on text generation (e.g., summarization, translation). Google initially prioritized BERT, while OpenAI focused on GPT, showcasing the architecture’s versatility. The use of large datasets to train Transformer models significantly advanced various NLP applications, including NER. Comparing zero-, one-, and five-shot learning approaches with these models offers valuable performance insights.

In this study, ChatGPT-4 turbo’s limited context window (8k–32k in the free interface, though 128k via paid API) posed a challenge. A 32k context proved insufficient for mammography reports, with inconsistencies emerging beyond 20k tokens. Gemini 1.5 Pro’s 1.5-million-token context window provided a significant advantage. Other large-context models like Claude (262k context window) offer similar benefits over ChatGPT’s free version, warranting further investigation. Early GPT-based NER research often used limited examples (0–5 shot prompts). This study’s findings, however, suggest that large-context LLMs, particularly with many-shot prompting, can significantly improve accuracy for non-English medical reports.

Current GPT models have limitations, notably processing time (60–90 s per report). Locally downloaded LLMs offer offline functionality and enhanced data privacy but often require substantial hardware resources and may exhibit reduced medical language proficiency. Prioritizing development and implementation of these local LLMs is crucial for applications requiring HIPAA/GDPR compliance.

The entity definitions used here are study-specific. For universal definitions, refer to RadGraph 1 [15] and RadGraph 2 [23], or consult established guidelines. RadGraph 2 introduced new entity types, many categorized as “impressions” in our study. Our lowest F1 scores were for uncertainty observations (OBS-U), potentially due to limited occurrences or underestimation of uncertainty indicators. Future refinements could involve extracting descriptive phrases within OBS-U entities and revising the definition. The RadGraph 2 appendix offers a valuable reference for entity definitions.

This study focused specifically on the development and evaluation of a novel prompt engineering approach for annotating mammography reports. While we demonstrated its effectiveness using Gemini 1.5 Pro, the prompt itself is designed to be adaptable to other LLMs. Future work could explore the performance of this prompt with a wider range of LLMs, including Mistral Large, LLaMA 3, and Claude 3/3.5, and conduct a more detailed comparison across different models, architectures, and cost structures. This would provide valuable insights into the prompt’s generalizability and potential for broader application in multilingual clinical NLP.

## 6. Conclusions

This study demonstrates the potential of LLMs, such as Gemini 1.5 Pro, combined with many-shot prompt engineering, for accurate and efficient automated clinical information extraction from non-English medical reports. Our findings suggest this approach offers a promising pathway for developing scalable solutions across various languages and medical specialties, potentially improving clinical workflows, research, and patient care. This approach offers significant benefits for non-English clinical text processing, particularly in languages like Turkish, German, Spanish, French, and Italian, where existing NLP tools are often limited.

This approach directly addresses the growing need for multilingual NLP solutions by enabling the analysis of non-English medical text without relying on translation, which can introduce errors and complexities. While this study focuses on Turkish, the methodology has the potential to be adapted to other languages, including those with different grammatical structures than English. The prompt engineering techniques described herein can be readily applied to other languages, encouraging further research in multilingual clinical NLP.

Future research should prioritize refining prompt engineering techniques for enhanced performance and adaptability. Investigating the generalizability of these models across different medical specialties, languages, and data types is also crucial. Finally, developing robust strategies for local LLM deployment is essential to address limitations associated with cloud-based access, including internet dependency and data privacy concerns. Collaborative efforts in these areas will be vital for realizing the transformative potential of LLMs in multilingual healthcare information management.

## Figures and Tables

**Table 1:** Relaxed match scores.

Calculated by Python Sklearn.metrics Library’s Classification Report Function Call					Calculated by Python Seqeval Library			
Entity	Precision	Recall	F1-Score	Support (tp + fn)	Precision	Recall	F1-Score	Support (tp + fn)
ANAT	0.99	1	0.99	785	0.99	1.00	0.99	785
IMP	1.00	0.98	0.99	345	1.00	0.98	0.99	345
O	1.00	0.99	0.99	1084	n/a	n/a	n/a	n/a
OBS-A	0.99	1.00	1.00	366	0.99	1.00	1.00	366
OBS-P	0.99	1.00	1.00	732	0.99	1.00	1.00	732
OBS-U	0.99	0.98	0.99	122	0.99	0.98	0.99	122
**Total**								2350
**Accuracy**			0.99	3434			0.99	2350
**Macro avg**	0.99	0.99	0.99	3434	0.99	0.99	0.99	2350
**Weighted avg**	0.99	0.99	0.99	3434	0.99	1.00	0.99	2350

**Table 2:** Relaxed match recognition confusion matrix.

Tr\Pr	ANAT	IMP	O	OBS-A	OBS-P	OBS-U	TP	FP	FN	TN
ANAT	784	0	0	0	0	1	784	11	1	2638
IMP	0	339	1	1	4	0	339	1	6	3088
O	11	0	1069	2	2	0	1069	2	15	2348
OBS-A	0	0	0	366	0	0	366	3	0	3065
OBS-P	0	0	0	0	732	0	732	6	0	2696
OBS-U	0	1	1	0	0	120	120	1	2	3311

**Table 3:** Exact match f1 scores and TP, FP, and FN values.

Calculated by Spacy Benchmark/Evaluate Command								
Entity	Precision	Recall	F1-Score	Support		TP	FP	FN
ANAT	0.89	0.87	0.88	812		710	87	102
IMP	0.80	0.78	0.79	390		307	75	83
OBS-A	0.92	0.95	0.94	215		206	16	9
OBS-P	0.79	0.77	0.78	925		719	191	206
OBS-U	0.83	0.81	0.82	112		91	18	21
**Accuracy**			0.71	2454	**Total**	2033	387	421
**Macro avg**	0.84	0.83	0.84	2454				
**Weighted avg**	0.84	0.82	0.83	2454				

## Data Availability

The prompt engineering approach, including excerpts from the prompt instructions, is detailed in the Methods section and the Supplementary Materials. The training and test datasets, consisting of anonymized Turkish mammography reports, are currently located in a private GitHub repository [20] to ensure data security during the review process. Upon acceptance of the manuscript for publication, this repository will be made public, allowing other researchers to access the data, reproduce the results, and build upon this work. Further inquiries can be directed to the corresponding author.

## Supplementary Materials

The following supporting information can be downloaded at https://www.mdpi.com/article/10.3390/bioengineering12020168/s1. Table S1: Task and entity definition sections and a selection of instructions from the first part of the prompt. Table S2: A selection of instructions defining the observation entities from the first part of the prompt. Table S3: A selection of instructions defining the impression entities from the first part of the prompt. Table S4: Some specific instructions from the first part of the prompt. Table S5: Selected examples from the second portion of the prompt.
