# Supplementary material for: Automated Extraction of Key Entities from Non-English Mammography Reports Using Named Entity Recognition with Prompt Engineering
Source: Bioengineering (Basel). 2025 Feb 10;12(2):168. doi: 10.3390/bioengineering12020168 (PMC11852152; doi:10.3390/bioengineering12020168)
Supplement: Supplementary file 1 [file bioengineering-12-00168-s001.zip › bioengineering-3424220-supplementary.pdf]

Supplementary Materials

Automated Extraction of Key Entities from Non-English Mammography Reports using Named Entity Recognition with Prompt Engineering

Zafer Akcali [1,2], Hazal Selvi Cubuk [3], Arzu Oguz [2], Murat Kocak [1], Aydan Farzaliyeva [2], Fatih Guven [3], Mehmet Nezir Ramazanoglu [2], Efe Hasdemir [2], Ozden Altundag [2] and Ahmet Muhtesem Agildere [3]

1. Baskent University, Faculty of Medicine, Department of Medical Informatics
2. Baskent University, Faculty of Medicine, Department of Internal Medicine, Division of Medical Oncology
3. Baskent University, Faculty of Medicine, Department of Radiology

* Correspondence: muratkocak25@gmail.com

Table S1. Some sections and a selection of instructions from the first part of the prompt.

Task
Your task is to generate an HTML version of a mammography report, marking up specific healthcare-related entities. The entities to be identified are: "anatomy", "observation-present", "observation-absent", "observation-uncertain", and "impression". Use HTML tags to highlight these entities, each with a class attribute indicating the type of the entity.
Output Format:
Always provide the output as an HTML code snippet enclosed within a code snippet window. Include style definitions for each entity class to visually differentiate them. Do not output as plain text.

When rendering the output, be sure to preserve the line breaks from the input text by including <br> tags after each line. This ensures the original formatting is maintained in the output.
Example:
Input:
This is line 1.
This is line 2.
Output:
This is line 1.
This is line 2.

Entity Definitions
1- Anatomy (ANAT): This category denotes regions of the human body, specifically referencing anatomical areas or organs. Examples include, but are not limited to:
* akciğer (lung)
* aksilla (axilla)
* aksiller kuyruk (axillary tail)
* aksiyel düzlemde (in the axial plane)
* alan (area)
* alanda (in the ... area)

Table S2. Some sections and a selection of instructions from the first part of the prompt.

2- Observations(OBS-P or OBS-A): This category encompasses specific findings, characteristics or medical devices observed in the medical report, including their descriptive modifiers. These modifiers provide crucial context about the observations, such as their quantity, prominence, distribution, or specific characteristics. Do not mark them inside an IMP tag. Examples include, but are not limited to:
Medical Findings:
* dansite (density)
* dansite artımları (density increases)
* dansite artışları (density increases)
* dansiteler (densities)
* değişiklikler (changes)
* distorsiyon (distortion)
* artmıştır (increased)
* asimetri (asymmetry)
Medical Findings with Descriptive Modifiers:
* nodüler kalınlaşma (nodular thickening)
* nodüler lezyon (nodular lesion)
* nonspesifik nodüller (nonspecific nodules)

Numerical Measurements: When annotating medical reports, pay close attention to numerical measurements and sizes associated with medical findings. These measurements are crucial for diagnosis and tracking the progression or regression of conditions. Always mark them as "observation-present" using the ... tag, except for dates. This includes a range of measurement formats:
* Millimeters (mm): e.g., "3 mm", "10x8 mm", "10 mm"

3- Observation-uncertain (OBS-U): Denotes an uncertain observation, where the condition or characteristic is not definitively present or absent. This includes individual words and phrases that convey uncertainty. Examples include, but are not limited to:
* grafiye dahil olmadığı düşünüldü (it was considered that they were not included in the field of view of the image)
* grafiye dahil olmayabileceği düşünüldü (it was considered that they might not be included in the field of view of the image)
* görüntü kalitesi nedeniyle kesin tanı konulamadı (definitive diagnosis could not be made due to image quality)
* ile uyumlu olabilecek (may be consistent with)
* izlenim vermekte (appears)

Table S3. Some sections and a selection of instructions from the first part of the prompt.

- Evaluations or descriptions of the condition's nature (IMP). Examples include, but are not limited to:
* BIRADS 0 (BIRADS category 0)
* BIRADS I (BIRADS category I)
* benign natürde (of a benign nature)
* benign radyolojik karakterde (with a benign radiological appearance)

-Phrases that describe the cause or reason for a finding (IMP). This category includes phrases that explain why a particular finding is present. These phrases often indicate that the finding is a result of a previous event, such as a surgery, procedure, or underlying condition. Examples:
* USG de tariflenen fibroadenom ile uyumludur (Consistent with the fibroadenoma described on ultrasound)

* ayırıcı tanı (differential diagnosis)
* bağlı (due to, associated with, secondary to)
- Patient's known history of malignancies, diseases or complaints (IMP), such as:
* ALL tanısı (diagnosis of acute lymphoblastic leukemia)
* aile hikayesi olan (with a family history of)
* akciğer Ca (lung carcinoma)
- Recommendations for further investigation (IMP). Examples include, but are not limited to:
* 6 ay sonra sağ unilateral MLO planda mamogramla korelasyonu önerilir (Correlation with mammography in the right unilateral MLO view is recommended in 6 months)
* US ile değerlendirilmesi önerilir (evaluation with ultrasound is recommended)
* değerlendirilmesi önerilir (it is recommended to evaluate)
* Dinamik meme MRG ile değerlendirme sonrası mamografi eşliğinde işaretleme ile eksizyonu önerilir (Excision with marking under mammography guidance is recommended after evaluation with dynamic breast MRI)
* ek incelemesi önerilmektedir (additional examination is recommended)
- Direct references to changes over time or treatment effects. This category includes phrases that describe how a finding has changed over time or in response to treatment (IMP). Examples include, but are not limited to:
* artış göstermiştir (has increased)
* artış gösterdiği (an increase is observed compared to)

Table S4. Some sections and a selection of instructions from the first part of the prompt.

Specific Instructions

Do not mark up the words as they are not medical entities, regardless of their location in the text:
* YORUM
* SONUÇ
* ÖNERİ
* IVKM

Do not mark up these terms as they are not medical entities, regardless of their location in the text:
diğer bulgularda (in the other findings), grafiye dahil olan (included in the graph), grafiye kısmen dahil olan (partially included in the graph), incelemeye dahil (included in the examination), noniyonik kontrast madde kullanılmıştır (non-ionic contrast agent has been used), önceki tetkikinde izlenmeyen (which were not visible on the previous examination), tanımlanan (defined), tariflenen (described), tarihli tetkikinden (compared to the examination dated), ve (and), veya (or), ya da (or), yaklaşık (approximately)

Annotation Guidelines
All reports are written in Turkish language. So do not translate Inputs and Outputs to English Language. All words are as is. Don't delete sentences.
###
Correct typographical errors before marking:
Incorrect: kalınlaşma bulguus izlenmedi.
Correct: kalınlaşma bulgusu izlenmedi.

Table S5. Selected examples from the second portion of the prompt.

Input: SONUÇ
Output: SONUÇ

Input: Sol meme parankimi dens (Tip D meme) görünümdedir<br>Output: Sol meme parankimi dens (Tip D meme) görünümdedir
Input: (Radyoterapi ve cerrahiye sekonder olarak değerlendirilmiştir)<br>Output: (Radyoterapi ve cerrahiye sekonder olarak değerlendirilmiştir)
Input: Sekretuar kalsifikasyona ait olabilir<br>Output: Sekretuar kalsifikasyona ait olabilir
Input: Sağ memede belirgin sınır veren kitle, nodül saptanmadı<br>Output: Sağ memede belirgin sınır veren kitle, nodül saptanmadı
Input: HASTANIN SAĞ MAMMOGRAFİ RAPORUNUN DA OKUNMASI ÖNERİLİR<br>Output: HASTANIN SAĞ MAMMOGRAFİ RAPORUNUN DA OKUNMASI ÖNERİLİR
Input: Grafiye dahil olan sol ön aksiller kesimde patolojik boyutta lenf nodu izlenmedi<br>Output: Grafiye dahil olan sol ön aksiller kesimde patolojik boyutta lenf nodu izlenmedi
